# Region-specific deletions of RIM1 reproduce a subset of global RIM1*α*^−/−^ phenotypes

**DOI:** 10.1111/j.1601-183X.2011.00755.x

**Published:** 2012-01-13

**Authors:** M E Haws, P S Kaeser, D L Jarvis, T C Südhof, C M Powell

**Affiliations:** 1 Department of Neurology and NeurotherapeuticsDallas, TX, USA; 2 Neuroscience Graduate ProgramDallas, TX, USA; 3 Department of Psychiatry, The University of Texas Southwestern Medical CenterDallas, TX, USA; 4 Department of Molecular and Cellular Physiology and Howard Hughes Medical Institute, Stanford UniversityPalo Alto, CA, USA

**Keywords:** CA3, cerebellum, cre recombinase, dentate gyrus, kainate, locomotor, MK-801, preproopiomelanocortin, RIM1, schizophrenia

## Abstract

The presynaptic protein RIM1*α* mediates multiple forms of presynaptic plasticity at both excitatory and inhibitory synapses. Previous studies of mice lacking RIM1*α* (RIM1*α*^−/−^ throughout the brain showed that deletion of RIM1*α* results in multiple behavioral abnormalities. In an effort to begin to delineate the brain regions in which RIM1 deletion mediates these abnormal behaviors, we used conditional (floxed) RIM1 knockout mice (fRIM1). By crossing these fRIM1 mice to previously characterized transgenic cre lines, we aimed to delete RIM1 selectively in the dentate gyrus (DG), using a specific preproopiomelanocortin promoter driving cre recombinase (POMC-cre) line , and in pyramidal neurons of the CA3 region of hippocampus, using the kainate receptor subunit 1 promoter driving cre recombinase (KA-cre). Neither of these cre driver lines was uniquely selective to the targeted regions. In spite of this, we were able to reproduce a subset of the global RIM1*α*^−/−^ behavioral abnormalities, thereby narrowing the brain regions in which loss of RIM1 is sufficient to produce these behavioral differences. Most interestingly, hypersensitivity to the pyschotomimetic MK-801 was shown in mice lacking RIM1 selectively in the DG, arcuate nucleus of the hypothalamus and select cerebellar neurons, implicating novel brain regions and neuronal subtypes in this behavior.

In mice, global deletion of presynaptic proteins often results in significant behavioral abnormalities reminiscent of human cognitive disease, though an understanding of brain regions or cell types mediating such phenotypes are not clear. For example, post-mortem studies on schizophrenic brains have consistently found alterations in presynaptic proteins, particularly genes involved in GABAergic neurotransmission, in the cerebellum, hippocampus and cortex (Akbarian and Huang 2006; Eastwood *et al.* 2001; Glantz & Lewis 1997; Guidotti *et al.* 2000; Sawada *et al.* 2005; Tamminga *et al.* 2004; Woo *et al.* 2004). Neurexin, another presynaptic protein, has been associated with autism (Etherton *et al.* 2009; Feng *et al.* 2006; Kim *et al.* 2008).

Recently, we have found that deletion of the presynaptic protein Rab3a interacting molecule 1*α* (RIM1*α*) in mice induced significant behavioral abnormalities, including some schizophrenia-relevant phenotypes, though RIM1*α* has not been directly implicated in human schizophrenia. These abnormalities include learning deficits (Powell *et al.* 2004), decreased prepulse inhibition (Blundell *et al.* 2010b), increased locomotor response to novelty (Powell *et al.* 2004), deficits in social interaction (Blundell *et al.* 2010b), increased sensitivity to the non-competitive N-methyl-D-aspartate receptor (NMDAR) antagonist MK-801 (Blundell *et al.* 2010b) and deficits in maternal behavior (Schoch *et al.* 2002). RIM1*α* was previously shown to express in most neurons (Schoch *et al.* 2006) and has been shown to mediate specific forms of presynaptic plasticity in hippocampal mossy fibers [dentate gyrus (DG) to CA3 synapses], Schaffer collaterals (CA3 to CA1 synapses), cerebellar parallel fibers (granule cell to purkinje synapses) and GABAergic interneurons (Calakos *et al.* 2004; Castillo *et al.* 2002; Chevaleyre *et al.* 2007; Schoch *et al.* 2002; Yang & Calakos 2010).

Given RIM1's diverse functions and expression, attributing behavioral phenotypes in the RIM1*α* knockout mouse to the loss of RIM1*α* from specific neurons is difficult. Consequently, we generated a conditional (floxed) RIM1 knockout (fRIM1) and crossed it to previously characterized transgenic cre recombinase lines. Initially, we aimed to examine the behavioral effects of deleting RIM1 selectively in DG granule neurons, where it is required for presynaptic long-term potentiation in mossy fibers (Castillo *et al.* 2002). Additionally, we aimed to examine the effect of RIM1 deletion selectively in area CA3 pyramidal neurons, where it mediates multiple forms of presynaptic plasticity (Calakos *et al.* 2004; Schoch *et al.* 2002). Unfortunately, neither cre driver line was as selective when crossed to our fRIM1 mutants as had been previously reported (Balthasar *et al.* 2004; McHugh *et al.* 2007; Nakazawa *et al.* 2002). Indeed, regional selectivity results of a reporter gene did not correlate completely with our regional measurements of RIM1 mRNA levels.

Nevertheless, we observed that a subset of behaviors were altered in these two regionally restricted RIM1 conditional deletion lines, thereby narrowing the brain regions involved in some behaviors. Specifically, mice lacking RIM1 selectively in the DG, arcuate nucleus of the hypothalamus and select neurons of the cerebellum resulted in increased sensitivity to the psychotomimetic drug MK-801. Our findings also suggest that loss of RIM1 in other brain regions or in multiple brain regions simultaneously may partially reproduce other behavioral abnormalities observed in RIM1*α*^−/−^ mice.

## Methods

### Genetic manipulations

Floxed RIM1 (fRIM1) mice for genetic removal of RIM1*α* and RIM1*β* were generated previously (Kaeser *et al.* 2008). Briefly, upon homologous recombination of the RIM1*αβ* targeting vector, 129Sv R1 stem cells containing the fRIM1 construct were injected into C57BL/6J blastocysts to produce chimeric mice, which were crossed for one generation to C57BL/6J for germline transmission. They were then crossed to flp recombinase mice (which were generated by injecting the flp transgene-containing vector into a fertilized egg from a B6SJLF_1_/J X B6SJLF_1_/J cross; Dymecki 1996) and the resultant offspring were intercrossed to generate the homozygous fRIM1 mice. The wild-type (WT), floxed, and recombined RIM1 alleles were genotyped by polymerase chain reaction (PCR) with oligonucleotide primers as described previously (Kaeser *et al.* 2008). The preproopiomelanocortin promoter driving cre recombinase (POMC-cre) mouse was generously provided by Joel Elmquist; it was generated in FVB/NJ mice and previously backcrossed three times to C57BL/6J as previously reported (Balthasar *et al.* 2004; McHugh *et al.* 2007). The kainate receptor subunit 1 promoter driving cre recombinase (KA-cre) mouse was generously provided by Susumu Tonegawa; it was generated in C57BL/6J mice, crossed to the Roas26 reporter mouse (Soriano 1999) and then subsequently backcrossed eight times to C57BL/6J mice as previously reported (Nakazawa *et al.* 2002). To generate the fRIM1/POMC-cre and fRIM1/KA-cre mice with sex-matched littermate controls, we used the following three-step breeding strategy. (1) The POMC-cre or KA-cre mice were crossed with fRIM1 homozygous mice. (2) The resulting fRIM1 heterozygous, cre positive mouse from cross 1 was crossed again with fRIM1 homozygotes. (3) The resulting fRIM1 homozygous, cre positive mouse from cross 2 was crossed again with fRIM1 homozygotes. This final cross provided sex-matched littermate pairs on a mixed genetic background that were fRIM1 homozygous, cre positive (fRIM1/cre+, experimental) and fRIM1 homozygous, cre negative (fRIM1/cre-, littermate control). Mice that displayed cre-mediated recombination in the tail DNA were excluded from our study.

### Immunohistochemistry

Following intracardial perfusion of fRIM1/POMC-cre+/ROSA-yellow flourescent protein (YFP) or fRIM1/KA-cre+/ROSA-YFP triple transgenic mice with 4% paraformaldehyde (Sigma, St Louis, MO, USA), whole brains were post-fixed in 4% paraformaldehyde at 4°C overnight. Brains were then transferred to 30% sucrose (Sigma) and allowed to equilibrate until they sank. Sections of 30-µm thickness were cut (HM 430 Sliding microtome, Microm; Waldorf, Germany) and stored at 4°C in phosphate-buffered saline (PBS) containing 0.1% sodium azide (Sigma) until use. Tissue sections were mounted onto positively charged glass slides (Fisher, Waltham, MA, USA) and allowed to air dry. A 0.1 m citric acid (Sigma) antigen unmasking treatment was performed before blocking slices with 3% normal donkey serum (NDS; Jackson ImmunoResearch, West Grove, PA, USA) in PBS containing 0.3% Triton-X-100 (Sigma). Overnight primary antibody incubation in 3% NDS, 0.3% Tween-20 (Sigma) in PBS at room temperature (chicken anti-GFP 1:2000, Invitrogen, Carlsbad, CA, USA; mouse anti-NeuN 1:250, mouse anti-parvalbumin 1:400 and rabbit anti-neurogranin 1:200, Millipore, Billerica, MA, USA) was followed by a 2-h secondary antibody treatment (biotin-conjugated donkey anti-chicken, CY3-conjugated donkey anti-mouse or CY3-conjugated donkey anti-rabbit; Jackson ImmunoResearch). Amplification was performed using the Avidin Biotin Complex Kit (Vector Laboratories, Burlingame, CA, USA) and a Tyramide Signal Amplification Kit (PerkenElmer, Waltham, MA, USA). Images were taken on an Olympus BX51 epifluorescent microscope (Tokyo, Japan).

### Quantitative reverse transcriptase polymerase chain reaction

Rapid tissue harvesting for quantitative reverse transcriptase PCR (qRT-PCR) was performed in chilled dissection solution [26 mm NaHCO_3_, 212.7 mm sucrose, 2.6 mm KCL, 1.23 mm NaH_2_PO_4_, 10 mm dextrose, 3 mm MgCl_2_•6(H_2_O), 1 mm CaCl_2_•2(H_2_O)] and stored at −80°C until mRNA was extracted with Trizol (Invitrogen). Reverse transcription of the mRNA library was performed using Superscript III 1st strand synthesis kit (Invitrogen). SYBR GreenER qPCR SuperMix Universal Kit (Invitrogen) was used for qRT-PCR according to the manufacturer's instructions. Each tissue sample was tested in triplicate and their Ct values were averaged together. Oligonucleotide primers were designed to create a 105-bp product spanning portions of exons 5 and 6 of the RIM1 mRNA transcript (forward primer – AAGCAGGCATCAAGGTCAAG, reverse primer – ACGTTTGCGCTCACTCTTCT). The neuron-specific microtubule-associated protein 2 (MAP2) was used as a reference gene (Mukaetova-Ladinska *et al.* 2002; Sundberg *et al.* 2006) (forward primer – ACTTGACAATGCTCACCACGTA, reverse primer – CCTTTGCATGCTCTCTGAAGTT). The relative change in RIM1 transcript between experimental and control tissue was normalized to MAP2 transcript levels using standard calculation methods (Pfaffl 2001). To test if the normalized relative change in RIM1 transcript levels was significantly different from unity (no change), a one-sample *t*-test was employed.

### Behavioral overview

Mice were housed two per cage (always littermate pairs) and maintained on a light/dark cycle with light on from 0600 to 1800 hours in a 22 ± 2°C (30–70% humidity) housing room. All behavioral tests were performed in the afternoon. For both fRIM1/POMC-cre+ and fRIM1/KA-cre+ mice, fRIM1/cre− sex-matched littermates were used as WT controls to ensure a constant genetic background. Food (normal chow) and water were available *ad libitum* for the duration of the behavioral battery. Sex-matched littermate pairs ranged from 3 to 8 months of age at the onset of behavioral testing and all behaviors were completed within 8 weeks. Two cohorts of fRIM1/POMC-cre (*N* = 12 and *N* = 8 littermate pairs, total *N* = 20 littermate pairs; littermate pairs age range at onset of behavior: 3 months, 3 pairs; 4 months, 3 pairs; 6 months, 10 pairs; 7 months, 1 pair; 8 moths, 3 pairs) and two cohorts of fRIM1/KA-cre (*N* = 10 and *N* = 10 littermate pairs, total *N* = 20 littermate pairs; littermate pairs age range at onset of behavior: 3 months, 5 pairs; 4 months, 4 pairs; 5 months, 4 pairs; 6 months, 3 pairs; 7 months, 4 pairs) were tested separately and pooled for analysis (except for rotarod, startle threshold, PPI and locomotor response to psychotomimetics in which only one of the cohorts was tested). In total, 13 male and 7 female fRIM1/KA-cre littermate pairs and 9 male and 11 female fRIM1/POMC-cre littermate pairs were tested. Behaviors were performed in the following order: elevated plus maze, dark/light, open field, locomotor, social interaction in the open field, accelerating rotarod, startle threshold, prepulse inhibition, fear conditioning, Morris water maze (MWM) and locomotor response to MK-801. Mice were allowed 1 h to habituate to the testing room before beginning experiments. All tests were carried out in accordance with Institutional Animal Care and Use Committee (IACUC) and UT Southwestern Medical Center animal guidelines and protocols.

#### Elevated plus maze

Elevated plus maze was performed essentially as described previously (Powell *et al.* 2004). Mice were placed in the center of a black plexiglass elevated plus maze (each arm 33 cm in length and 5 cm wide, with 25-cm-high walls on closed arms) in a dimly lit room for 5 min. Two mazes were used and video-tracked simultaneously (Ethovision 2.3.19, Noldus, Wageningen, The Netherlands). A barrier was set between the mazes to prevent mice from seeing each other. Time spent in open and closed arms, number of open and closed entries, and time in the middle was calculated. Five fRIM1/POMC-cre and one fRIM1/KA-cre pairs were excluded from analysis because either the experimental or control mouse fell from the platform during testing.

#### Dark/light

Performed essentially as described previously (Powell *et al.* 2004). Apparatus is a two-compartment opaque plexiglass box (25 × 26 cm in each compartment). One side is black and kept closed and dark, while the other is white with a fluorescent light directly above its open top (1700 lx). Mice were placed in the dark side for 2 min, then the divider between the two sides was removed allowing the mouse to freely explore both chambers for 10 min. Anxiety-like behavior is measured as latency to enter the light side, as well as time spent in the light versus dark compartments. Measures were taken using photobeams and MedPC software (Med Associates, St Albans, VT, USA).

#### Open field

Performed essentially as described previously (Powell *et al.* 2004). The open field test was performed for 10 min in a brightly lit, 48 × 48 × 48 cm white plastic arena using the Ethovision video-tracking software (Noldus). Time spent in the center zone (15 × 15 cm) and frequency to enter the center was recorded. Locomotor activity was also measured during the open field test.

#### Locomotor

Mice were placed in a fresh home cage with minimal bedding for a 2-h testing period. Lengthwise horizontal locomotor activity was tested in 5-min bins for the duration of the task using photobeams linked to computer data acquisition software (San Diego Instruments, San Diego, CA, USA). Beams were organized linearly along one horizontal axis in 5-cm increments and total beam breaks were used as the dependent variable.

#### Social interaction in the open field

Performed essentially as described previously (Blundell *et al.* 2010a). The test was performed in a 48 × 48 cm white plastic arena under red light using a 6.0 × 9.5 cm perforated plexiglass rectangular cage containing an unfamiliar adult mouse, allowing olfactory, visual, and minimal tactile interaction. Mice were first placed in the arena for 5 min with the empty clear plexiglass cage. Then mice were allowed to interact with a novel caged social target for another 5 min. Time spent in the interaction zone was obtained using Ethovision video-tracking software (Noldus).

#### Accelerating rotarod

Performed essentially as described previously (Powell *et al.* 2004). A five-lane accelerating rotarod designed for mice (IITC Life Science, Woodland Hills, CA, USA) was used (rod diameter was 3.2 cm; rod length was 10.5 cm). The rotarod was activated after placing mice on the motionless rod. The rod accelerated from 0 to 45 revolutions per min in 60 seconds. Time to fall off the rod was measured.

#### Startle threshold and prepulse inhibition

Both these tasks were performed as described previously (Etherton *et al.* 2009). The *prepulse inhibition* (PPI) test began with six presentations of a 120-dB pulse to calculate the initial startle response. Afterward, the 120-dB pulse alone, prepulse/pulse pairings of 4, 8 or 16 dB above 70-dB background followed by the 120-dB pulse with a 100-millisecond delay or no stimulus were presented in pseudorandom order. For the *startle threshold* test, mice were presented with six trial types of varying intensity (no stimulus or 80-, 90-, 100-, 110- or 120-dB pulses – eight presentations of each). Mean startle amplitudes for each condition were averaged.

#### Fear conditioning

Performed essentially as described previously (Powell *et al.* 2004). Mice were placed in clear plexiglass shock boxes (Med Associates) for 2 min, and then two 90-dB acoustic conditioned stimuli (CS; white noise, each 30 seconds in duration and separated by a 30-second delay) were played. Each CS co-terminated in a 2-second, 0.5-mA foot shock (US). Mice remained in the chamber for 2 min after the second pairing before returning to their home cages. Freezing behavior (motionless except respirations) was monitored at 5-second intervals by an observer blind to the genotype. To test contextual learning 24 h later, mice were returned to the same training context and scored for freezing in the same manner. To assess cue-dependent fear conditioning, mice were placed in a novel environment with an unfamiliar vanilla odor in the afternoon following the contextual test. Freezing was measured first during a 3-min baseline period then during 3 min with the CS playing.

#### Morris water maze

The MWM and visible platform tests were performed as described previously (Powell *et al.* 2004) except the probe trial was performed only on day 9. Briefly, a 1.22-m-diameter, white, plastic, circular pool was filled with opaque water (22 ± 1°C) in a room with prominent extra-maze cues. Mice were trained with four trials per day with an intertrial interval of 1–1.5 min for 8 consecutive days. Mice were placed in one of four starting locations and allowed to swim until they found the submerged platform or until a maximum of 60 seconds had elapsed. Latency to reach the platform, distance traveled to reach the platform, swim speed and percent thigmotaxis (time spent near the wall of the pool) were measured using the Ethovision video-tracking software (Noldus). A probe trial (free swim with the submerged platform removed) was performed on day 9. The percent time spent in the target quadrant and the number of platform location crossings were measured. The *visible water maze* task was conducted similarly to the traditional MWM with a few changes. A visible cue (black foam cube) was placed on top of the platform. The starting location was held constant, while the platform location was moved for each trial. Mice were given six trials per day for 2 consecutive days, and the latency to reach the visible platform was measured.

#### Locomotor response to psychotomimetics

The apparatus and methods described for the locomotor test were also used to measure locomotion in this task. The test was performed as described by Blundell *et al.* (2010b). Briefly, at the beginning of each hour of a 3-h locomotor session, mice were given a ∼0.2 ± 0.1 cc intraperitoneal injection of saline (time 0), 0.1 mg/kg MK-801 (1 h) and 0.2 mg/kg MK-801 (2 h). Because fRIM1/KA-cre+ showed increased locomotion to novel environments, they underwent 3 days of this protocol (receiving only saline injections) to habituate before the actual testing on day 4.

### Statistics

We used genotype and sex as independent variables for all behavioral tests and performed two-way analyses of variance (anovas) for statistical analysis. Where applicable, time (locomotion tasks), trial (rotarod and visual MWM tasks), day (MWM training) or intensity (PPI and startle threshold) were included as independent variables and a three-way anova with repeated measures was performed. In the MWM probe trial, genotype, sex and location were included as independent variables and a three-way anova was performed. A Student's *t*-test was used for the planned comparisons analysis in the locomotor tasks and the MWM probe trial. We used Statistica (StatSoft, Inc., Tulsa, OK, USA) for statistical analysis and significance was always taken as *P* < 0.05.

## Results

### fRIM1/POMC-cre+ results in RIM1 deletion in DG, arcuate nucleus and select neurons of the cerebellum

We first crossed fRIM1 mice to the POMC-cre transgenic line. This particular POMC-cre line was previously reported to induce recombination selectively in DG granule neurons as well as the arcuate nucleus of the hypothalamus (Balthasar *et al.* 2004; McHugh *et al.* 2007). Recombination was reported to begin at 2–3 weeks of age and to remain spatially restricted into adulthood. Assessing the location of RIM1 knockout is particularly challenging due to the absence of antibodies that function selectively in immunohistochemistry (IHC) and the high degree of homology between RIM1 and RIM2. Thus, we first crossed in the previously described conditional Rosa reporter transgenic line (R26R-YFP) (Lagace *et al.* 2007; Soriano 1999) to obtain fRIM1/POMC-cre+/R26R-YFP mice to label cre recombinase activity in the presence of fRIM1.

**Figure 1:**
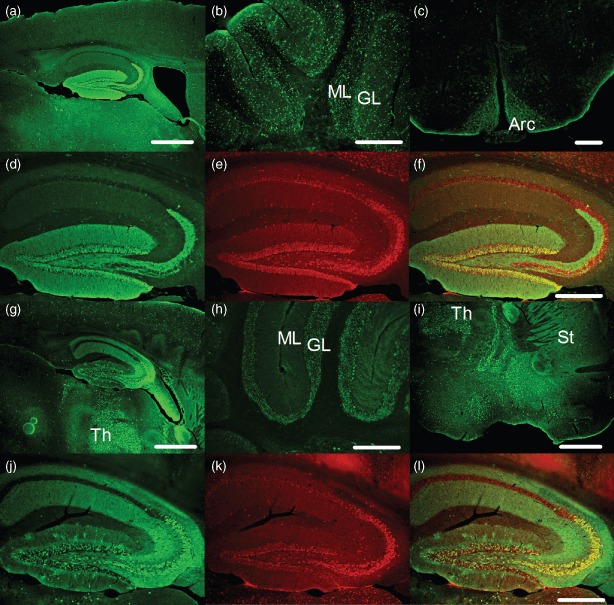
Immunofluorescent signal shows regional specificity of the fRIM1/POMC-cre+/R26R-YFP and fRIM1/KA-cre+/R26R-YFP reporter mice YFP expression in fRIM1/POMC-cre+/R26R-YFP brain tissue from hippocampus (a), cerebellum (b) and hypothalamus (c). YFP and NeuN expression in a fRIM1/POMC-cre+/R26R-YFP hippocampus (d–f). FP expression in fRIM1/KA-cre+/R26R-YFP brain tissue from hippocampus (g), cerebelleum (h) and forebrain (i). YFP and NeuN expression in a fRIM1/KA/R26R-YFP hippocampus (j and k). f and l are merges of d and e and j and k, respectively (green – anti-YFP, red – anti-NeuN; a, h and i, scale bar = 1 mm; b, d–f, h, j and k, scale bar = 500 µm; c, scale bar = 250 µm; ML, molecular layer; GL, granule layer; Th, thalamus; St, striatum).

IHC staining for YFP in the fRIM1/POMC-cre+/R26R-YFP mice showed successful cre-mediated recombination largely limited to the DG granule neurons, select neurons in the granule and molecular layers of the cerebellum, and arcuate nucleus of the hypothalamus (Figure 1a–c). Co-staining for the neuronal marker NeuN showed that cre-mediated recombination in DG neurons was robust, but somewhat mosaic. Interneurons in the stratum radiatum, stratum lacunosum-moleculare and stratum lucidum of the hippocampus and in the hilus region did not undergo cre-mediated recombination, nor did pyramidal neurons in area CA3 or CA1 (Figure 1d–f).

**Figure 2:**
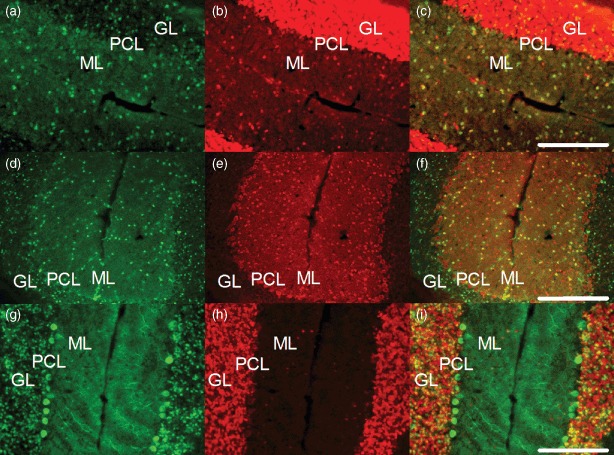
Cell-specific cre-mediated recombination in fRIM1/POMC-cre+/R26R-YFP and fRIM1/KA-cre+/R26R-YFP cerebellum (a–c) High magnification of YFP and NeuN expression in the cerebellum of a fRIM1/POMC-cre+/R26R-YFP mouse. (d–f) High magnification of YFP and parvalbumin expression in the cerebellum of a fRIM1/POMC-cre+/R26R-YFP mouse. (g–i) High magnification of YFP and NeuN expression in the cerebellum of a fRIM1/KA-cre+/R26R-YFP mouse. c, f and i are merges of a and b, d and e and g and h, respectively (green – anti-YFP, red – anti-NeuN or anti-parvalbumin as labeled; all scale bars = 250 µm; GL, granule layer; PCL, purkinje cell layer; ML, molecular layer).

The YFP staining pattern in cerebellum suggested cre-mediated recombination restricted to neurons of the granular and molecular layers without involvement of purkinje cells (Figure 2a–c). In the molecular layer, consisting largely of interneurons, this was confirmed by co-staining for parvalbumin-containing interneurons showing that almost all YFP-positive neurons in this layer were also positive for the inhibitory interneuron marker parvalbumin, though cre-mediated recombination did not occur in all parvalbumin positive neurons (Figure 2d–f). The cerebellar granular layer largely consists of granule neurons making mosaic staining for YFP in this region most likely to be in granule cells. To rule out Golgi cells, another common neuron type in the granule cell layer (GL), as responsible for the YFP expression in the GL, we co-stained for the Golgi cell marker neurogranin (Singec *et al.* 2003). Essentially none of the YFP positive cells co-stained for neurogranin (data not shown), making it most likely that the YFP-positive neurons in this region represent mosaic cre recombination in granule cells of the cerebellum.

**Figure 3:**
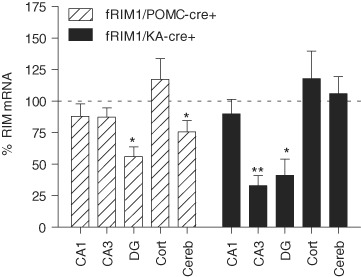
Regional specificity of fRIM1/POMC-cre+ and fRIM1/KA-cre+ conditional knockouts by qRT-PCR Quantitative RT-PCR was used to measure levels of RIM1 transcript in the CA1, CA3 and dentate gyrus regions of the hippocampus as well as cortex and cerebellum in the fRIM1/POMC-cre+ and fRIM1/KA-cre+ mice compared with their littermate controls. RIM1 transcript levels were internally normalized to MAP2 transcript levels and reported as a percentage of normalized RIM1 transcript levels observed in littermate controls (dashed line; *P < 0.05; **P < 0.01).

To address whether levels of RIM1 transcript were decreased in YFP+ brain regions, we performed qRT-PCR on fRIM1/POMC-cre+ and fRIM1/POMC-cre− control tissue from cortex, cerebellum and three hippocampal regions: DG, CA3 and CA1 (Figure 3). Consistent with cre-mediated recombination data, we found RIM1 transcripts to be decreased by 43.89% in the DG (SEM = ±7.48%, *P* < 0.05) and 24.39% in the cerebellum (SEM = ±8.97%, *P* < 0.05) in fRIM1/POMC-cre+ mice compared with controls.

### fRIM1/KA-cre+ results in RIM1 deletion in CA3 pyramidal neurons as well as mosaic deletion in DG and other brain regions

We next crossed fRIM1 mice to the KA-cre line. This KA-cre line was previously shown to selectively induce recombination in CA3 pyramidal neurons, 10% of DG granule neurons and cerebellar granule neurons, as well as 50% of facial nerve nuclei of the hindbrain (Nakazawa *et al.* 2002). In area CA3, recombination was first observed at 2 weeks of age with 100% of CA3 pyramidal neurons recombined by 8 weeks. Again, we crossed in the previously described conditional Rosa reporter transgenic line (R26R-YFP; Lagace *et al.* 2007; Soriano 1999) to generate fRIM1/KA-cre+/R26R-YFP mice, which would label cre recombinase activity in the presence of fRIM1.

IHC staining for YFP in the fRIM1/KA-cre+/R26R-YFP mice showed robust cre-mediated recombination in area CA3 of the hippocampus and mosaic recombination in DG, cortex, thalamus and cerebellum (Figure 1g–i). We did not observe robust staining in the facial nerve nuclei (data not shown). Co-staining for the neuronal marker NeuN showed that cre-mediated recombination in virtually all CA3 pyramidal neurons, hilus region neurons and in a small fraction of DG granule neurons (Figure 1j–l). Interneurons in the stratum radiatum and stratum lacunosum-moleculare of the hippocampus and area CA1 pyramidal neurons did not undergo cre-mediated recombination (Figure 1j–l). We also observed YFP staining in cerebellar purkinje cells and mosaically in neurons of the cerebellar granule layer (Figure 2g–i).

Using qRT-PCR, we measured RIM1 transcript levels in the fRIM1/KA-cre+ mice in the same five brain regions analyzed in the fRIM1/POMC-cre+ mice (Figure 3). RIM1 transcript levels were decreased by 58.86% in the DG (SEM = ±12.83%, *P* < 0.05) and 67.02% in area CA3 (SEM = ±7.96%, *P* < 0.05) of fRIM1/KA-cre+ mice compared with controls.

### POMC-cre-mediated loss of RIM1 in DG, selected cerebellar neurons and arcuate nucleus leads to increased locomotor responses to the psychotomimetic MK-801

Previous work showed that RIM1*α*^−/−^ mice had severe behavioral abnormalities that included some schizophrenia-related deficits (Blundell *et al.* 2010b), learning and memory deficits (Powell *et al.* 2004), decreased maternal behavior (Schoch *et al.* 2002) and social interaction abnormalities (Powell *et al.* 2004). To test whether loss of RIM1 from the DG, select neurons of the cerebellum and arcuate nucleus of the hypothalamus in the fRIM1/POMC-cre+ mice was sufficient to induce any of the behavioral abnormalities observed in the RIM1*α*^−/−^ mice, we put the fRIM1/POMC-cre+ mice with sex-matched littermate controls through many of the same behavioral tasks.

**Figure 4:**
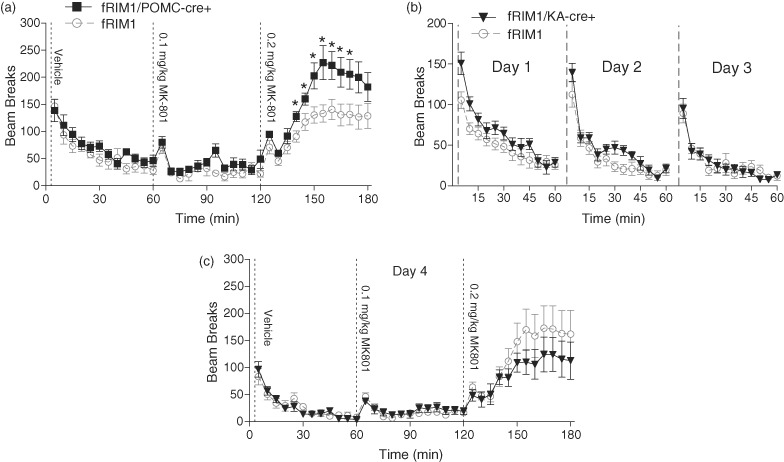
fRIM1/POMC-cre+ mice exhibit enhanced locomotor response to psychotomimetics (a) Three-hour locomotor test in which fRIM1/POMC-cre+ mice received an injection of saline, 0.1 mg/kg MK-801 or 0.2 mg/kg MK-801 at the beginning of each hour as marked by the vertical dotted lines. (b) As fRIM1/KA-cre+ mice are hyperactive in novel environments, they were habituated to the 3-h locomotor test over 3 days, receiving only saline injections at the start of each hour (only the first hour is shown). (c) On day 4, fRIM1/KA-cre+ mice underwent the 3-h locomotor test, receiving saline, 0.1 mg/kg MK-801 or 0.2 mg/kg MK-801 at the start of each hour as marked by the vertical dotted line (*P < 0.05 using Student's *t*-test).

When we tested the locomotor enhancing effects of MK-801, we found that the fRIM1/POMC-cre+ mice mimicked the global RIM1*α*^−/−^. Specifically, there is no change in locomotion when injected with saline (Figure 4a; three-way mixed anova with repeated measures; main effect of genotype, *F*_1,12_ = 1.09*,P* = 0.32; main effect of time, *F*_11,132_ = 15.76, *P* < 0.0001; main effect of sex, *F*_1,12_ = 0.004*,P* = 0.95; genotype × time interaction, *F*_11,132_ = 0.60*,P* = 0.83; genotype × sex interaction, *F*_1,12_ = 0.38*,P* = 0.55; time × sex interaction, *F*_11,132_ = 0.60*,P* = 0.83; genotype × time × sex interaction: *F*_11,132_ = 0.74*,P* = 0.69) and no change in locomotion at the low 0.1 mg/kg MK-801 dose (Figure 4a; main effect of genotype, *F*_1,12_ = 1.93*,P* = 0.19; main effect of time, *F*_11,132_ = 5.11*,P <* 0.0001; main effect of sex, *F*_1,12_ = 1.03*,P* = 0.33; genotype × time interaction, *F*_11,154_ = 0.84*,P* = 0.60; genotype × sex interaction, *F*_1,12_ = 5.05*,P <* 0.05; time × sex interaction, *F*_11,132_ = 0.80*,P* = 0.64; sex × genotype × bin interaction, *F*_11,132_ = 0.68*,P* = 0.75). However, there is a significant interaction between genotype and time during the hour following the higher 0.2 mg/kg MK-801 dose showing that the response to this higher dose of MK-801 is different between the two genotypes (Figure 4a; main effect of genotype, *F*_1,12_ = 4.39*,P* = 0.06; main effect of time, *F*_11,132_ = 22.02*,P <* 0.0001; main effect of sex, *F*_1,12_ = 0.13*,P* = 0.73; genotype × time interaction, *F*_11,132_ = 2.07*,P <* 0.05; genotype × sex interaction, *F*_1,12_ = 0.002*,P* = 0.99; time × sex interaction, *F*_11,132_ = 0.20*,P* = 0.997; genotype × time × sex interaction, *F*_11,132_ = 0.38*,P* = 0.96). Planned comparison analysis identified that mutant mice were significantly different from littermate controls following the 0.2 mg/kg MK-801 injection. These planned comparisons indicated that fRIM1/POMC-cre+ mice are significantly more active beginning 20 min after injection (*P* < 0.05) and begin returning toward control levels of locomotion 50 min following the injection.

**Figure 5:**
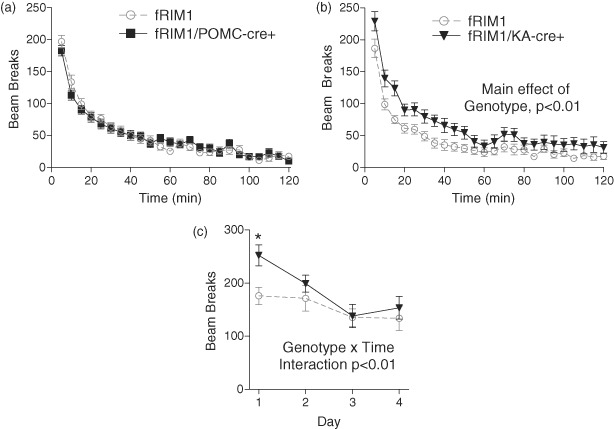
fRIM1/KA-cre+ mice display enhanced locomotion to novel stimuli (a) fRIM1/POMC-cre+ mice underwent a 2-h test of locomotion in a novel home cage in which lengthwise movement was monitored using photobeams. (b) fRIM1/KA-cre+ mice underwent the same test. (c) Locomotor habituation of the fRIM1/KA-cre+ mice: Over 4 days, the first 10 min of locomotor activity was recorded (*P < 0.05 using Student's *t*-test).

**Figure 6:**
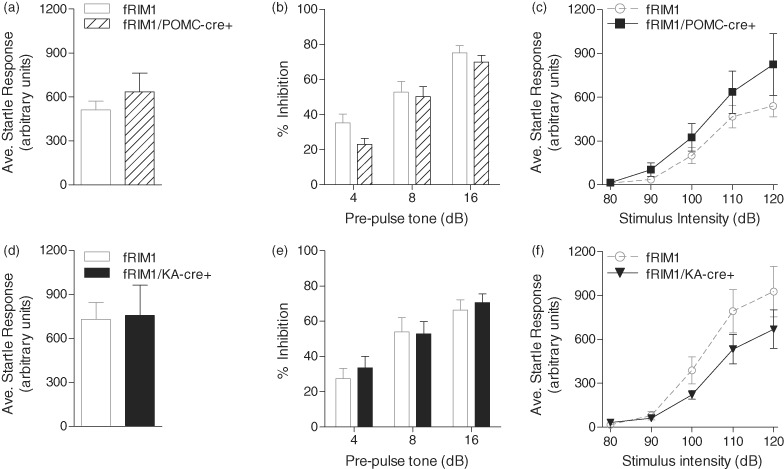
fRIM1/POMC-cre+ and fRIM1/KA-cre+ mice display normal startle response and prepulse inhibition (a and d) The average startle response to a 120-dB tone of fRIM1/POMC-cre+ and fRIM1/KA-cre+ mice, respectively, before the start of the prepulse inhibition test. (b and e) Prepulse inhibition test. The percent decrease in the startle response caused by a 4-, 8- or 16-dB prepulse tone given 100 milliseconds before the 120-dB stimulus tone. (c and f) Startle threshold. Average startle response to increasing stimulus intensity delivered in a pseudo random order.

**Figure 7:**
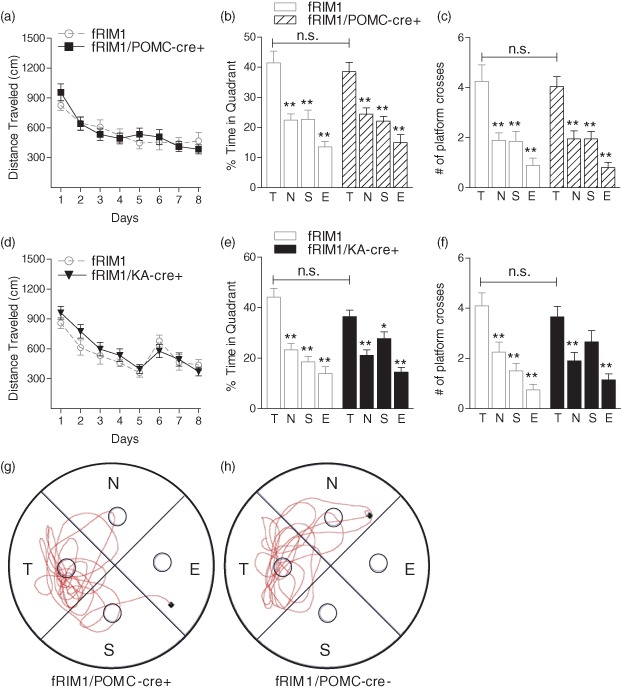
fRIM1/KA-cre+ and fRIM1/POMC-cre+ display normal spatial learning in the MWM (a) During 8 days of training in the Morris water maze, the distance traveled to find the hidden platform was measured in the fRIM1/POMC-cre+ mice. (b and c) fRIM1/POMC-cre+ probe trial. On day 9, the platform was removed from the pool and the amount of time spent in each quadrant (b) and the number of platform location crossings (c) was recorded. (d) Eight-day training period in Morris water maze of the fRIM1/KA-cre+ mice. (e and f) fRIM1/KA-cre+ probe trial. The platform was removed from the pool and the amount of time spent in each quadrant (e) and the number of platform location crossings (f) was recorded. (g and h) Representative tracings from the MWM probe trial of fRIM1/POMC-cre+ and fRIM1/POMC-cre− mice, respectively (quadrants and platform locations: T, target (west); N, north; S, south; E, east; *P < 0.05, *P < 0.01; n.s., not significant).

**Figure 8:**
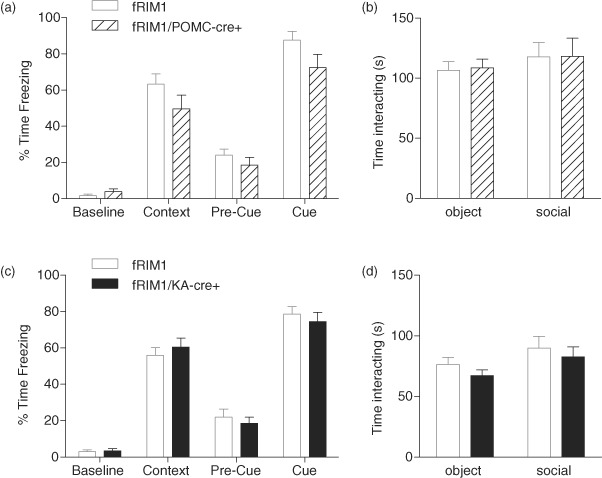
Contextual and cued fear conditioning as well as social interaction in the open field are normal in both the fRIM1/POMC-cre+ and fRIM1/KA-cre+ mice (a and c). Fear conditioning in fRIM1/POMC-cre+ and fRIM1/KA-cre+ mice, respectively. Baseline freezing was measured during the first 2 min in the novel context before tone/footshock delivery. Contextual fear conditioning was measured during a 5-min exposure to the same context the following day. Cued fear conditioning was also measured the following day. Mice were exposed to a different context without the tone for 3 min (pretone), followed by 3 min with the tone playing (tone). (b and d) Social interaction in the open field was measured as the amount of time spent in an interaction zone with either an empty cage (object) or a cage containing a target mouse (social).

**Figure 9:**
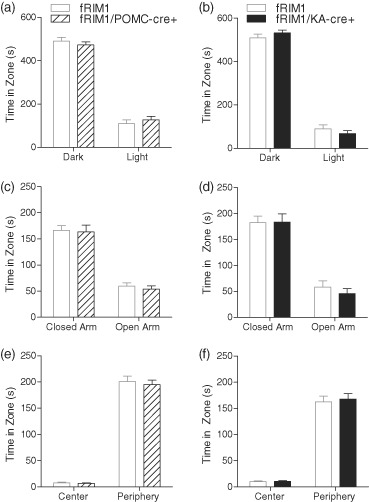
Three measures of anxiety are normal in both the fRIM1/POMC-cre+ and fRIM1/KA-cre+ mice (a and b) Dark/light. The amount of time spent in the dark versus light zones is reported. (c and d) Elevated plus maze. The amount of time spent in the open versus closed arms is reported. (e and f) Open field. The amount of time spent in the center versus periphery of the open field box is reported.

When we tested the fRIM1/POMC-cre+ mice for other abnormalities found in the RIM1*α*^−/−^ mice, including locomotor activity to novelty (Figure 5a), PPI (Figure 6b), MWM (Figure 7a–c), fear conditioning (Figure 8a) and social interaction (Figure 8b), we observed no differences compared with littermate controls. We also tested these mice for changes in startle response (Figure 6a and c), and anxiety-related behaviors (dark/light box, elevated plus maze and open field; Figure 9a, c and e, respectively) and accelerating rotarod (data not shown), but found no differences in any of these behaviors compared with littermate controls.

### KA-cre-mediated loss of RIM1 in CA3 of hippocampus and mosaic loss in DG and other brain regions leads to hyperactivity to novelty

We tested the fRIM1/KA-cre+ mice with sex-matched littermate controls in the same behavioral tasks as the fRIM1/POMC-cre+ mice. fRIM1/KA-cre+ mice mimicked RIM1*α*^−/−^ mice only in their hyperactivity to novelty. Specifically, fRIM1/KA-cre+ mice were hyperactive during a 2-h test of locomotion (Figure 5b; three-way mixed anova; main effect of genotype, *F*_1,36_ = 7.79*,P <* 0.01; main effect of time, *F*_23,828_ = 61.68*,P <* 0.001; main effect of sex, *F*_1,36_ = 0.48*,P* = 0.49; genotype × time interaction, *F*_23,828_ = 1.17*,P* = 0.27; genotype × sex interaction, *F*_1,36_ = 0.47*,P* = 0.50; time × sex interaction, *F*_23,828_ = 0.63*,P* = 0.91; genotype × time × sex, *F*_23,828_ = 0.38*,P* = 0.997). When we evaluated the first 10 min of locomotor activity in the same environment over 4 days, their locomotor activity diminished until it was equal to littermate controls (Figure 5c; three-way mixed anova; main effect of genotype, *F*_1,16_ = 0.52*,P* = 0.48; main effect of day, *F*_3,48_ = 13.44*,P <* 0.0001; main effect of sex, *F*_1,16_ = 0.05*,P* = 0.83; genotype × day interaction, *F*_3,48_ = 3.12*,P <* 0.05; genotype × sex interaction, *F*_1,16_ = 0.30*,P* = 0.59; day × sex interaction, *F*_3,48_ = 1.67*,P* = 0.19; genotype × day × sex interaction, *F*_3,48_ = 1.34*,P* = 0.27; Student's *t*-test was used for a planned comparison on day 1, *P* < 0.05). Similar to RIM1*α*^−/−^ mice, to test the locomotor response to MK-801 in the fRIM1/KA-cre+ mice, we had to habituate the fRIM1/KA-cre+ mice to the MK-801-induced locomotor task protocol for 3 days using only saline injections (Figure 4b). On the fourth day, we followed the same MK-801-induced locomotor activity protocol performed on the fRIM1/POMC-cre+ mice. In this case, however, we saw no difference between fRIM1/KA-cre+ mice and littermate controls (Figure 4c), providing a double dissociation between locomotor response to novelty (in fRIM1/KA-cre+ but not fRIM1/POMC-cre+ mice) and locomotor response to the psychotomimetic MK-801 (in fRIM1/POMC-cre+ but not fRIM1/KA-cre+ mice). We also tested the fRIM1/KA-cre+ mice in startle threshold and PPI (Figure 6d–f), MWM (Figure 7d–f), fear conditioning (Figure 8c), social interaction in the open field (Figure 8d) anxiety tests (Figure 9b, d and f) and accelerating rotarod (data not shown). In all these tests, the fRIM1/KA-cre+ mice were not significantly different from littermate controls.

## Discussion

We have previously shown that global deletion of RIM1*α* results in multiple behavioral abnormalities, including learning deficits in fear conditioning (Powell *et al.* 2004) and MWM (Powell *et al.* 2004), decreased prepulse inhibition (Blundell *et al.* 2010b), increased locomotor response to novelty (Powell *et al.* 2004), deficits in social interaction (Blundell *et al.* 2010b), increased sensitivity to the non-competitive NMDAR antagonist MK-801 (Blundell *et al.* 2010b) and deficits in maternal behavior (Schoch *et al.* 2002). The present results serve to narrow the brain regions responsible for the increased sensitivity to the non-competitive NMDAR antagonist MK-801 and the increased locomotor response to novelty (Table 1). Deficits in maternal behavior and pup rearing were not examined in the present study as they would require specific, complex breeding strategies outside the scope of the present studies.

**Table 1:** Summary of behavioral deficits in the RIM1*α*^−/−^, fRIM1/POMC-cre+ and fRIM1/KA-cre+ mice

Behavior	RIM1*α*^−/−^	fRIM1/POMC-cre+	fRIM1/KA-cre+
Elevated plus maze	Normal	Normal	Normal
Open field	Not tested	Normal	Normal
Dark/light	Normal	Normal	Normal
Locomotion	Hyperactivity to novelty	Normal	Hyperactivity to novelty
Social interaction with a juvenile	Decreased social interaction	Not tested	Not tested
Social interaction in the open field	Not tested	Normal	Normal
Rotarod	Normal	Normal	Normal
Startle threshold	Not tested	Normal	Normal
Prepulse inhibition	Decreased prepulse inhibition	Normal	Normal
Contextual and cued fear conditioning	Decreased freezing	Normal	Normal
Morris water maze	Did not learn to find platform	Normal	Normal
Locomotor response to MK-801	Enhanced locomotor response	Enhanced locomotor response	Normal
Maternal behavior	Decreased maternal behavior	Not tested	Not tested

### Characterization of fRIM1/POMC-cre+ and fRIM1/KA-cre+ mice

The fRIM1/POMC-cre+ and fRIM1/KA-cre+ mice lack RIM1 in distinct, partially overlapping brain regions. fRIM1/POMC-cre+ mice exhibit selective cre-mediated recombination in DG granule neurons, arcuate nucleus of the hypothalamus, PV+ GABAergic interneurons of the cerebellar molecular layer (Yu *et al.* 1996) and scattered neurons of the cerebellar granule layer. This pattern of cre-mediated recombination in fRIM1/POMC-cre+ mice is supported by our qRT-PCR data showing significantly decreased RIM1 mRNA expression levels in both DG and cerebellum, but not in other areas of the hippocampus or the cortex. fRIM1/KA-cre+ mice show robust cre-mediated recombination in area CA3 of the hippocampus and mosaic recombination in hippocampal DG granule neurons, granule and purkinje layers of the cerebellum, thalamus and cortex. Again, the most robust cre-mediated recombination in fRIM1/KA-cre+ mice in CA3 and DG was confirmed by qRT-PCR, while the mosaic, cre-mediated recombination in other parts of the brain was not supported by our qRT-PCR data, suggesting that cre-mediated recombination measured by a reporter transgene does not always accurately reflect cre-mediated recombination of a different conditional allele. The RIM1 loss in the cerebellum of fRIM1/POMC-cre+ mice and the widespread mosaic expression of cre-mediated recombination in brains from fRIM1/KA-cre+ mice differ from previous reports characterizing the POMC and KA-cre driver lines (Balthasar *et al.* 2004; McHugh *et al.* 2007; Nakazawa *et al.* 2002). Changes in expression patterns of cre recombinase due to changes in genetic background, however, have been observed in other cre driver lines (Smith 2011). These findings underscore the importance of determining the regions of cre-mediated recombination in each experimental setting.

Although the fRIM1/POMC-cre+ mice and fRIM1/KA-cre+ mice show some overlapping cre expression, the two mouse lines do not share any behavioral abnormalities. This provides evidence that at least some of the abnormal behaviors observed in the RIM1*α*^−/−^ mice are because of a loss of RIM1 function from one or a few select classes of neurons. Identifying how alterations in presynaptic function in select neuronal subtypes alter select behaviors is important for understanding the cellular basis for these behaviors.

### Limited neuronal populations sufficient to induce increased MK-801-induced hyperactivity

Like the RIM1*α*^−/−^ mice, the fRIM1/POMC-cre+ mice exhibit hypersensitivity to the locomotor enhancing effects of MK-801 (Blundell *et al.* 2010b). Little is known about the specific brain regions or cell types involved in the locomotor effects of MK-801. Because the fRIM1/POMC-cre+ mice mimic the RIM1 mice, however, our results suggest that RIM1 function in the DG, arcuate nucleus or select neurons of the cerebellum (including PV+ interneurons and granule cells) may modulate the effect of MK-801 on locomotor activity. Although some or all of these regions may in fact play a role in this behavioral phenotype, one interesting possibility is a role for PV+ GABAergic interneurons. Studies on PV+ neurons in the cortex suggest that MK-801 acts preferentially on NMDA receptors expressed on PV+ GABAergic cells leading to decreased inhibition (Beasley & Reynolds 1997; Benes & Berretta 2001; Cochran *et al.* 2002; Hashimoto *et al.* 2003; Kinney *et al.* 2006; Lewis *et al.* 2005; Li *et al.* 2002; Xi *et al.* 2009). Unfortunately, we are unable to further narrow the brain regions or neuronal types involved in this phenotype due to the technical limitation that there are not selective RIM1 antibodies that work for IHC.

While both fRIM1/POMC-cre+ and fRIM1/KA-cre+ mice lead to cre-mediated recombination in the DG, only fRIM1/POMC-cre+ mice exhibit increased sensitivity to MK-801. At first glance, this would appear to rule out a role for loss of RIM1 in DG in this behavioral abnormality. The percentage of dentate granule neurons in which cre-mediated recombination occurs, however, is clearly much larger in fRIM1/POMC-cre+ mice than in fRIM1/KA-cre+ mice. Thus, we cannot rule out that an effect of loss of RIM1 in a majority of dentate granule neurons (fRIM1/POMC-cre+) rather than a smaller percentage (fRIM1/KA-cre+) may account for the increased sensitivity to MK-801 in fRIM1/POMC-cre+ mice. It is also possible that loss of RIM1 in combinations of the neuronal subtypes identified in fRIM1/POMC-cre+ is leading to the increased susceptibility to MK-801. Finally, though these mice performed normally in the rotarod task, we cannot rule out the possibility that other cerebellar-related abnormalities may become evident if a more extensive coordination test battery were performed.

### Lack of memory deficits in fRIM1/POMC-cre+ and fRIM1/KA-cre+ mice

Unlike RIM1*α*^−/−^, the fRIM1/POMC-cre+ and fRIM1/KA-cre+ mice did not show a learning deficit in hippocampus-dependent forms of learning and memory, including the MWM and fear conditioning. As fRIM1/POMC-cre+ lack RIM1 from most DG granule neurons, it suggests that RIM1-mediated forms of plasticity in DG granule neurons are either not required or that only a small percentage of RIM1 expressing DG granule neurons are necessary for these forms of learning. Similarly, because fRIM1/KA-cre+ mice lack RIM1 from almost 100% of CA3 pyramidal neurons, it seems that RIM1-dependent plasticity in area CA3 is not required for these forms of learning. It may also be that robust loss of RIM1 from multiple synapses in the hippocampal tri-synaptic pathway is necessary to induce significant learning and memory deficits. As fRIM1/KA-cre+ mice lack RIM1 robustly in area CA3 and mosaically in the DG, it raises the possibility that the learning and memory deficit observed in RIM1*α*^−/−^ mice may be due, at least in part, to the loss of RIM1 from extra-hippocampal synapses and may or may not require a loss of RIM1 in the hippocampus.

### fRIM1/KA-cre+ mice have a subtle phenotype compared with RIM1*α*^−/−^ mice

RIM1*α*^−/−^ mice exhibited increased locomotor activity to novelty (Blundell *et al.* 2010b). The fRIM1/KA-cre+ mice follow the same pattern except the increased locomotion is lesser in magnitude. Because of a lack of region selectivity and mosaicism of cre expression in fRIM1/KA-cre+ mice, it is difficult to pinpoint brain regions responsible for the increased locomotion to novelty. One interesting point suggested by our results, however, is the double dissociation between increased locomotion to novelty and enhanced MK-801-induced hyperactivity. These two phenotypes do not seem to be mediated by the same set of neurons.

### Overview

Overall, both the fRIM1/POMC-cre+ and fRIM1/KA-cre+ mice recapitulate limited components of the phenotypes observed in the RIM1*α*^−/−^ mice. Both these cre recombinase driver lines have been used in a variety of studies (Banno *et al.* 2010; Fukushima *et al.* 2009; Ramadori *et al.* 2010; Zhang *et al.* 2009), some of which specifically compared locomotor activity or swim speed in B6 mice with and without the cre transgene and no abnormalities were observed (Tonegawa *et al.* 2003; Xu *et al.* 2008). The phenotypes observed in the fRIM1/KA-cre+ mice are subtle and difficult to attribute to RIM1 loss from particular neurons. The hypersensitivity to locomotor activation effects of MK-801 was more narrowly limited to a few cell types and brain regions in the fRIM1/POMC-cre+ mice. One way to better study these brain regions in the future may be to use virally expressed cre recombinase to target specific brain regions without the lack of selectivity that often accompanies cre driver lines.
